# The impact of structured higher-order interactions on ecological network stability

**DOI:** 10.1007/s12080-025-00603-0

**Published:** 2025-01-29

**Authors:** J. Christopher D. Terry, Michael B. Bonsall, Rebecca J. Morris

**Affiliations:** 1 Department of Biology, University of Oxford, 11a Mansfield Road, Oxford, OX1 3SZ UK; 2 School of Biological Sciences, University of Southampton, University Road, Southampton, SO17 1BJ UK

**Keywords:** Interaction modification, Food web, Stability, Feasibility, Interaction network, Non-trophic effect, Higher-order interaction

## Abstract

The impact of higher-order interactions, those involving more than two species, is increasingly appreciated as having the potential to strongly influence the dynamics of complex ecological systems. However, although the critical importance of the structure of pairwise interaction networks is well established, studies of higher-order interactions still largely assume random structures. Here, we demonstrate the strong impact of structured higher-order interactions on simulated ecological communities. We focus on effects caused by interaction modifications within food webs, where a consumer resource interaction is modified by a third species, and for which plausible structures can be hypothesised. We show how interaction modifications introduced under a range of non-random distributions may impact the overall network structure. Local stability and the size of the feasibility domain are critically dependent on the inter-relationship between trophic and non-trophic effects. Where interaction modifications are structured into mutual interference motifs (associated with consumers switching between resources) synergistic signs and topological effects have particularly consequential impacts. Furthermore, we show that previous results of the impact of higher-order interactions on diversity-stability relationships can be reversed when higher-order interactions are structured, not random. Empirical data on interaction modifications will be a key part of improving understanding the dynamics of communities, particularly the distribution of interaction modification signs across networks.

**Supplementary Information:**

The online version contains supplementary material available at 10.1007/s12080-025-00603-0.

## Introduction

Explorations of how the structures of interaction networks influence the dynamics of ecosystems have been central to the development of ecology (Dunne and Pascual 2006; McCann 2011; Moore and de Ruiter 2012). Theoretical expectations that large random complex systems are unlikely to be locally stable (May 1972) have led to a search for features within ecological interaction networks that can mitigate this instability (Montoya et al. 2006; Thébault and Fontaine 2010). In particular, an extensive body of work has demonstrated features of trophic networks that can stabilise communities—including the distribution of weak links (McCann et al. 1998; Neutel et al. 2002), pairwise correlations (Tang et al. 2014), modularity (Grilli et al. 2016), row structure (Jacquet et al. 2016) and trophic level coherence (Johnson and Jones 2017). However, ecological communities are made up of multiple complex networks of interactions beyond pairwise trophic relationships (Ings et al. 2009). There is an emerging appreciation that studying the full spectrum of interaction types simultaneously can bring additional insight (Coyte et al. 2015; Fontaine et al. 2011; García-Callejas et al. 2018; Kéfi et al. 2012; Miele et al. 2019; Olff et al. 2009; Pilosof et al. 2017).

**Fig. 1:**
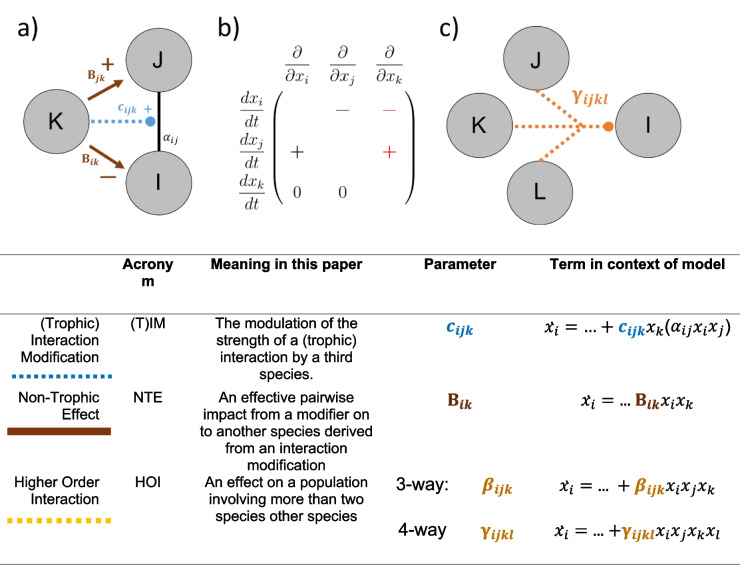
Clarification of key terms. **a** Schematic representation of the relationship between a facilitatory trophic interaction modification (TIM, blue dashed line) and consequent two non-trophic effects (NTE, brown solid lines). We label this TIM ‘facilitatory’ (rather than ‘interfering’) because an increase in the modifier species increases the strength of the interaction. **b** Resultant community matrix, where each column shows the effect of an increase in a species on the growth rate of species in each row (i.e. ${{\varvec{A}}}_{{\varvec{i}}{\varvec{j}}}=\frac{\partial }{\partial {x}_{j}}\frac{d{x}_{i}}{dt}$), where ${x}_{i}$ is the abundance of species $i$. Here, an interaction modification results in two non-trophic effects (in red). The modifier species $k$ acts to strengthen a trophic interaction between consumer $j$ on resource $i$. This results in a positive NTE on $j$ and a negative NTE on $i$. **c** An example 4-way higher-order interaction where species $j$, $k$ and $l$ have a combined impact on the dynamics of focal species $i$

A notable fraction of non-trophic effects are caused by interaction modifications (Kéfi et al. 2012), a type of higher-order interaction where the strength of a pairwise interaction is dependent on a third species (Billick and Case 1994; Terry et al. 2017; Wootton 1994b, a). These ‘higher-order’ processes induce non-trophic effects from the modifier species onto a pair of interactors (Fig. 1). Interaction modifications are pervasive within ecological networks (Werner and Peacor 2003) and capable of exerting impacts as strong as direct trophic interactions (Preisser et al. 2005). Their theoretical impact on small community modules has been widely studied (Bolker et al. 2003; Křivan 2014; Ohgushi et al. 2012) and the incorporation of interaction modification effects has been empirically demonstrated to be necessary to understand the fitness of competing species (Mayfield and Stouffer 2017), community persistence (van Veen et al. 2005), and the response of communities to species loss (Donohue et al. 2017). Classic examples of trophic interaction modifications include behavioural shifts in foraging patterns in response to the threat of predators (Pringle et al. 2019; Suraci et al. 2016) and species providing associational resistance to predation (Barbosa et al. 2009). However, the concept encompasses any biotic influences on foraging patterns, such as the availability of alternative prey (Abrams 2010) and the diverse array of ecosystem engineering effects (Sanders et al. 2014). It is also increasingly recognised within microbial systems, where antibiotic degradation by third-species can attenuate inhibition between species (Kelsic et al. 2015).

This additional source of interactions introduces emergent relationships between species that may not otherwise directly interact, greatly increasing the overall connectance of the system (Yodzis 2000). The incorporation of randomly distributed higher-order interactions (HOIs) has been shown to have substantial impacts on community stability (AlAdwani and Saavedra 2019; Bairey et al. 2016; Gibbs et al. 2022; Grilli et al. 2017b; Wilson 1992). The assumption of random HOIs has allowed the development of sophisticated formal analytical tools (Gibbs et al. 2022). However, the assumption of ‘random’ unstructured distributions can generate impacts distinct to any particular dynamic consequences from their ‘higher order’ nature. For example, Bairey et al. (2016) demonstrate a stabilising effect of random 4-way HOIs stemming from the reduction in the variance of effective pairwise interactions—when increasing numbers of random zero-centred HOI elements are summed over more dimensions, they cancel out more frequently and the resultant pairwise effects are increasingly likely to be weaker and less destabilising.

Although the true distribution of HOIs at the network level is essentially unknown, it is reasonable to expect that interaction modifications will be structured, both with respect to each other and with respect to the underlying interactions being modified (Golubski et al. 2016; Kéfi et al. 2016). Both trophic and non-trophic interactions can be subject to modifications (Kéfi et al. 2012). Although networks of many HOIs in diverse communities have been more frequently studied in ‘horizontal’ communities defined by competition and facilitation (Gibbs et al. 2022; Grilli et al. 2017b; Letten and Stouffer 2019; Levine et al. 2017; Mayfield and Stouffer 2017), individual interaction modifications have been most studied within trophic networks, particularly around the behavioural modifications from predator avoidance or prey switching (Ohgushi et al. 2012). A focus on trophic interaction modifications therefore offers an opportunity to build upon an understanding of the structure of trophic networks (Dunne and Pascual 2006; Moore et al. 2017) to explore the possible consequences of structured HOIs on community stability.

Here we conduct two analyses using simulated communities. Firstly, we investigate the impact of a series of structured distributions of trophic interaction modifications on overall network structure and stability around assumed equilibrium points. Secondly, we examine if a positive diversity-stability relationship found when simulating dynamics with unstructured higher-order interactions (Bairey et al. 2016) can be reproduced when the structure is introduced.

## Methods

### Terminology

A consistent brake on research beyond pairwise interactions has been the highly varied terminology, with both distinct interpretations of terms between papers and multiple labels applied to the same process (Abrams 2001; Kleinhesselink et al. 2022; Letten and Stouffer 2019; Terry et al. 2017). Distinguishing between the underlying trophic interaction modification (TIM) processes, the resultant (pairwise) non-trophic effects (NTEs, also termed *trait-mediated indirect interactions* (Ohgushi et al. 2012)), and higher order interaction (HOI) terms is a particular cause for confusion*.* For clarity, the meaning of key terms, acronyms and notation used here is described in Fig. 1. Here, trophic interaction modifications are a specific subset of possible higher-order interactions, while non-trophic effects are their representation in a community matrix.

### Generating structured higher-order interactions

Although databases of trophic networks have been established for many years (Cohen 1978; Poisot et al. 2016), empirical distributions of interaction modifications are essentially unknown (although see Kéfi et al. 2015) and likely to significantly differ between communities. We therefore developed a suite of models to examine the range of properties that such networks may have in order to identify how interaction modifications may introduce additional structure into ecological networks.

We determined the underlying trophic topology of the artificial communities using the niche model (Williams and Martinez 2000), a network-generating algorithm that has been shown to reproduce many of the features of real food webs (Williams and Martinez 2008). We parameterised each trophic interaction withdraws from a bivariate Gaussian distribution $\mathcal{N}\left({\mu }_{RC}=-1,{\mu }_{CR}=1,{sd}_{RC}=0.5,s{d}_{CR}=0.5,\rho =-0.8\right)$. This simple approach generates a negative correlation between the impact of the consumer on the resource and the resource on the consumer, in line with empirical observations (Allesina et al. 2015). We generated 100 trophic networks each with 60 species and target connectance of 0.2 to represent moderately large, fairly well-connected communities.

**Fig. 2:**
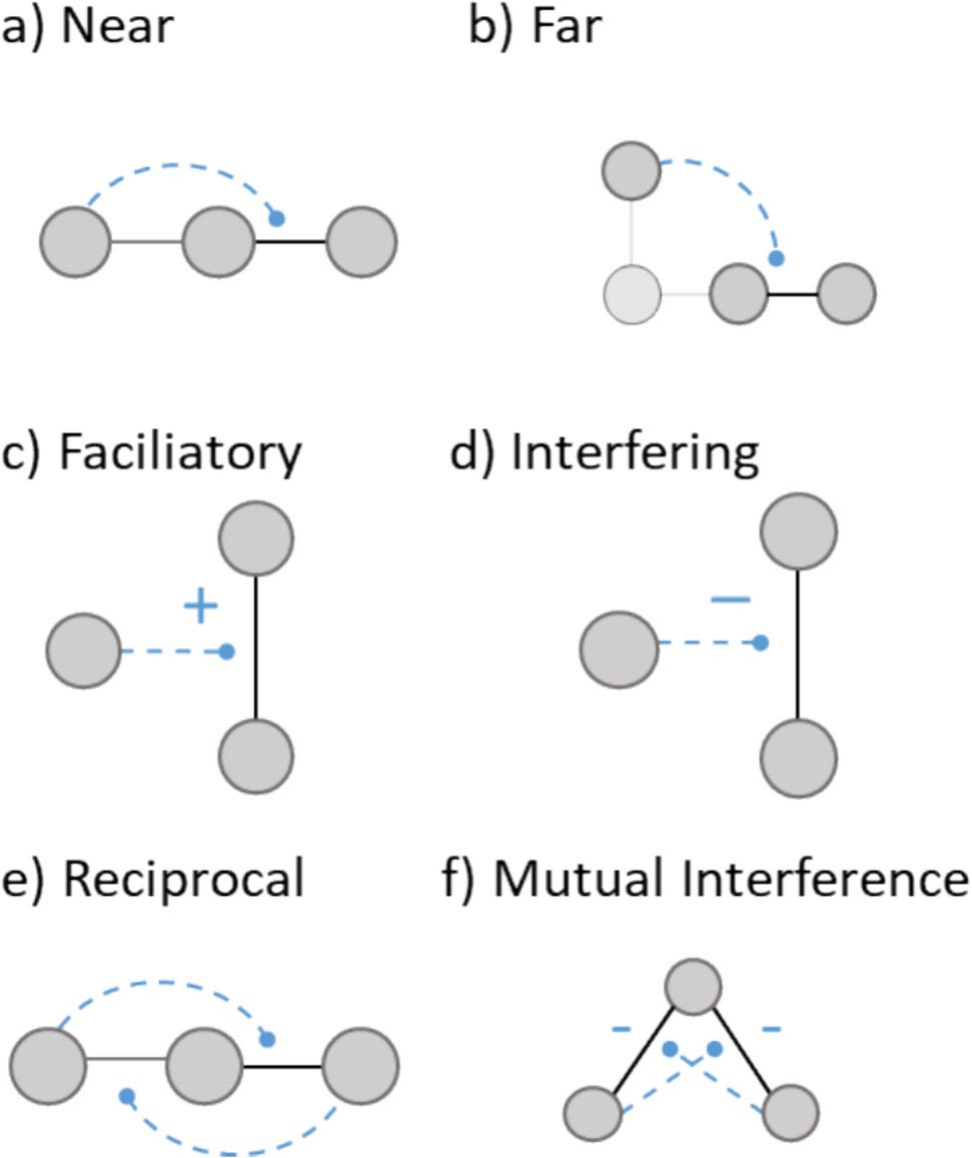
Illustration of the structured TIM distribution models used in this study. Cartoons illustrate the distinctive properties of each model compared to the baseline model that introduced interaction modifications such that each potential modification was equally and independently likely to occur (see the main text for a full description of each model)

Building upon these trophic networks, we tested seven distributional models (one random, six structured) for identifying the location and sign of TIMs relative to the underlying trophic network (Fig. 2). Trophic interaction modifications are defined by the identity of the three species involved (modifier, consumer, resource) and whether an increase in the modifier species would strengthen or weaken the trophic interaction. In the random baseline model, each possible interaction modification (combination of consumer, resource and third-party modifier species) has an equal likelihood of existing and has a random sign.

The non-random distribution models effectively generate distinctive network motifs (Milo et al. 2002) within the community. Two models introduced a simple dependence on the trophic distance between the modifier species and the trophic interactors: the ‘nearby-only’ model (Fig. 2a) required that the modifier was trophically connected to at least one of the interactors, while the ‘far-only’ model (Fig. 2b) excluded such cases. The sign of the modification was varied in two models, introducing either exclusively facilitating (Fig. 2c) or interfering (Fig. 2d) TIMs. A reciprocal model (Fig. 2e) introduced TIMs only in tightly reciprocal pairs to represent the clustering of interaction modifications within particular subgroups of species. In this model, sets of three trophically linked species include two reciprocal modifications—a species that modifies an interaction between species $i$ and species $j$ would in turn have its interaction with $j$ modified by $i$. Lastly, a widespread subset of interaction modifications are caused by foraging choices between two resources, where each resource reduces the consumption of the other by the shared consumer. We represented this with a ‘mutual interference’ model (Fig. 2f) where only negative reciprocal modifications that fit this description were introduced.

### Building effective interaction matrices

**Fig. 3:**
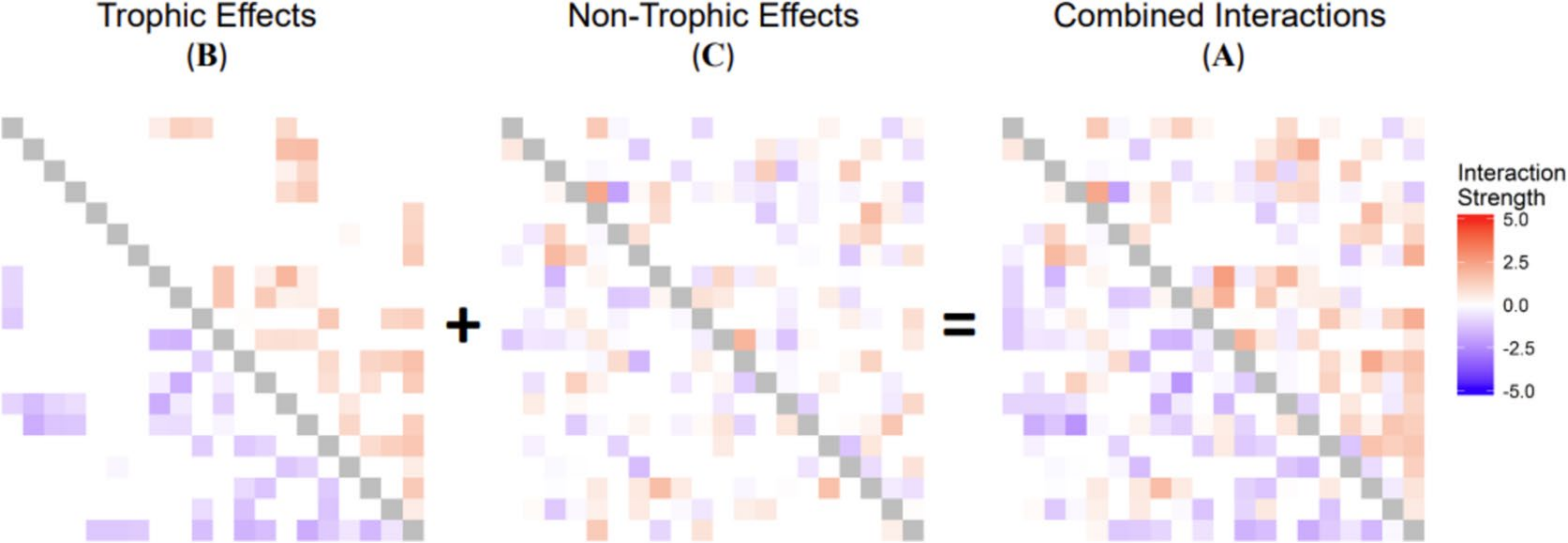
Illustration of construction of community matrices that specify the linearized interactions between species as quantified by the impact on population growth rates. At an assumed equilibrium, a combined community matrix ($\mathbf{A}$) is constructed from the sum of the impact of trophic ($\mathbf{B}$**)** and non-trophic effects ($\mathbf{C}$). Here, the species are arranged approximately in trophic height order, with basal species in the first rows and columns, and top predators in later rows and columns. The underlying trophic network depicted was generated with the niche model (Williams and Martinez 2000) with species number = 20, target connectance = 0.2 and parameterised with a bivariate Gaussian distribution. Intra-specific interactions are shown here in grey

To explore the impact of non-random interaction modifications on the structure and stability of communities, we represent the complete set of interactions in a system as a Jacobian matrix $\mathbf{A}$. Specifically, this is a community matrix (Novak et al. 2016) assumed to be derived from a set of populations each at a feasible equilibrium. Each element ${\mathbf{A}}_{{\varvec{i}}{\varvec{j}}}$, represents the total instantaneous effect of a change in the population of species $j$ on the population growth rate of species $i$. Although interaction modifications act through at least three species, the short-term consequences can be linearized to identify the effect of the modifier on the consumer and the resource (Bairey et al. 2016; Wilson 1992) at the system state under consideration (Fig. 1b). These non-trophic effects (NTEs) can be used to construct a matrix $\mathbf{C}$ of linearized pairwise effects caused by the TIMs. We build $\mathbf{A}$ from a matrix $\mathbf{B}$ specifying the trophic interactions and a matrix $\mathbf{C}$ specifying the non-trophic effects present in the community (Fig. 3).

### Building interaction matrices with structured interaction modifications

Based upon the set of 100 trophic networks, NTE matrices were generated using the seven TIM distribution models described above. TIMs were introduced at a target total frequency per species ($\lambda$) from 0 to 30, in increments of 2. We only considered TIMs where species modified the interaction between two other species—we did not allow species to modify their own interactions. Note that TIM frequency as defined here scales with, but is distinct to, both ‘TIM connectance’ (the fraction of possible TIMs that are observed, which is dependent on the trophic connectance) and to ‘non-trophic connectance’ (the resultant fraction of non-zero elements $\mathbf{C}$) which is dependent on the distribution and overlap of the TIMs). Note also that when motifs add two TIMs at a time each TIM is counted separately (see SI Figure S1 for examples of non-trophic effect matrices generated with each distribution model).

Each TIM was assigned an effect parameter, ${c}_{ijk}$, representing the size and direction of the modification by species $k$ of the consumption of resource $i$ by consumer $j$. A positive ${c}_{ijk}$ indicates a facilitating modification where an increase in the modifying species would increase the strength of the interaction. It follows that an increase in such a modifier species would lead to a positive non-trophic effect of that species on the consumer and a negative non-trophic effect of the modifier on the resource (Fig. 1). An interfering modification, where ${c}_{ijk}$ is negative, would cause the reverse. Values for ${c}_{ijk}$ were drawn from a Gaussian distribution of mean 0 and standard deviation $\alpha \sqrt{\pi }/\sqrt{2}$, where $\alpha$ is the target mean magnitude of individual NTEs. The standard deviation is scaled to maintain the mean magnitude of non-trophic effects as half the mean trophic interaction strength, in line with results from meta-analyses that suggest an approximate correspondence between the strength of trophic and non-trophic interactions (Preisser et al. 2005). Where a species modified more than one interaction with another species, the consequent non-trophic effects were combined additively.

### Properties of combined interaction matrices

We applied each of the distribution models at each TIM frequency across each of the 100 underlying trophic networks. As a further baseline, an approach adding a corresponding number of randomly distributed NTEs was also tested in which elements of $\mathbf{C}$ were independently randomly distributed with their strengths drawn from a Gaussian distribution. For each community, we calculated structural properties of the resultant interaction matrices that have been established as influencing aspects of dynamic stability: connectance (fraction non-zero entries) of $\mathbf{A}$ and $\mathbf{C}$, variance (V) of the off-diagonal elements $\mathbf{C}$, degree heterogeneity of $\mathbf{A}$ as the variance of the normalised in and out-degree distribution, correlation ($\uprho$) of the pairwise elements $\left(i.e. {\mathrm{A}}_{ij},{\mathrm{A}}_{ji}\right)$ within $\mathbf{A}$, and lastly the covariance between elements of trophic and non-trophic effects $\mathrm{cov}(\mathbf{B},\mathbf{C})$.

We calculated two measures of system dynamics for each community based on the overall matrix $\mathbf{A}$ described below—the local asymptotic stability and the size of the feasibility domain. Both these measures assume that the community described by $\mathbf{A}$ is at equilibrium and any perturbations are small enough that the collapse of the HOIs into an effective pairwise matrix is a reasonable approximation.

Local asymptotic stability is determined by the sign of the real part of the leading (dominant) eigenvalue of $\mathbf{A}$, $\mathfrak{R}\left({\uplambda }_{1}^{A}\right)$, under the assumption that the community is at a feasible equilibrium. If negative, the system will eventually return to the original equilibrium after a small perturbation and is considered locally stable. The diagonal elements of $\mathbf{A}$ specify the self-regulation of each species and with sufficient self-regulation, any community can be stabilised. Although the distribution of self-regulation effects can have important consequences (Barabás et al. 2017), we follow previous work (Allesina et al. 2015; Jacquet et al. 2016) and assume all diagonal elements of $\mathbf{A}$ to be zero in order to focus on the impact of the inter-specific interactions. Without self-regulation, $\mathfrak{R}\left({\uplambda }_{1}^{\mathrm{A}}\right)$ will always be positive but can be interpreted as how far a system is from stability, i.e. how much self-regulation would be necessary to stabilise the system. Hence, although local stability is a binary property, we use ‘less stable’ to refer to a system with a larger $\mathfrak{R}\left({\uplambda }_{1}^{\mathrm{A}}\right)$.

The size of the feasibility domain of a community is related to the set of environmental conditions under which all species have positive abundances (Saavedra et al. 2017; Song et al. 2018). Assuming that a community is feasible to begin with, it can be used to indicate structural stability in terms of the tolerance to variation in intrinsic growth rates (caused by environmental conditions) without a species being driven extinct (Rohr et al. 2014). A measure of the size of this feasibility domain, $\Omega \left(\mathbf{A}\right)$, can be derived from the probability that a randomly sampled vector of intrinsic growth rates allows all species to exist given a set of interactions specified by solely by a matrix $\mathbf{A}$. This can then be scaled to calculate the average probability that a randomly chosen species can feasibly exist (i.e. have a positive equilibrium density) as $\upomega \left(\mathbf{A}\right)=\Omega {\left(\mathbf{A}\right)}^\frac{1}{S}$. We calculated $\Omega \left(\mathbf{A}\right)$ following the method of Song et al. (2018). In contrast to local stability, feasibility is related in a non-trivial way to the values of the diagonal of the matrix being analysed (Grilli et al. 2017a). We therefore test the sensitivity of results to assigning all intra-specific interactions to be −2 but also test values of either 0 or − 5.

### Diversity-stability relationship under higher-order interactions

We next examined if an observed reversal of diversity-stability relationships for random higher-order interactions (Bairey et al. 2016) also held true for structured HOIs. Following Bairey et al. (2016), we built Lotka-Volterra class models that included pairwise, three-way HOIs and four-way HOIs:

$$\frac{dx_i}{dt}\frac1{x_i}=r_i\;\underbrace{-x_i}_{self-regulation}+\underbrace{\sum_{j=1}^N\sqrt\alpha A_{ij}\;x_j}_{Pairwise}\;+\;\underbrace{\sum_{j=1}^N\sum_{k=1}^N\sqrt\beta\;B_{ijk}\;x_j\;x_k}_{3-way\;HOIs}\;+\underbrace{\sum_{j=1}^N\sum_{k=1}^N\sum_{l=1}^N\sqrt\gamma C_{ijkl}\;x_j\;x_k\;x_l\cdot}_{4-\;way\;HOIs}$$

The model assumes $N$ species each with an abundance ${x}_{i}$ and showing self-regulation. The intrinsic growth of each species, ${r}_{i}$, was set to $1/N$. $A$, $B$ and $C$ are arrays with 2, 3 and 4 dimensions, respectively with elements drawn from a Gaussian distribution with a fixed mean of 0 and variance of 1. The interaction strength of the array of interactions is scaled by the square root of $\alpha$, $\upbeta$ and $\upgamma$ terms respectively, following Bairey et al. (2016).

In contrast to our earlier analyses, a positive equilibrium was not assumed. Instead, the feasibility of a given system was tested numerically by initiating each population at an abundance of 1/N, then integrating the system until either a species went extinct (falling below 0.0001/N) or stability was reached. Increasing the mean interaction strength will eventually lead to a loss of feasibility. The critical strength of an interaction order was defined as the highest strength where > 90% of 50 random communities showed no extinctions.

We tested how this critical strength depended on $N$ (ranging from 10 to 30) for a variety of scenarios. Firstly, we reproduced the results of Bairey et al. (2016), using random interaction arrays solely of pairwise, three-way or four-way interactions drawn from Gaussian distributions. Secondly, we then introduced a baseline pairwise trophic interaction network (with $\alpha$ = 0.001, and the variance of the elements further scaled by $1/\sqrt{N}$ to maintain a constant impact on stability) and tested introducing random three-way or four-way higher-order interactions. This tests if the underlying trophic network structure is influential. Thirdly, we again introduced a baseline pairwise trophic interaction network but tested introducing structured three-way or four-way higher-order interactions specified from trophic interaction modifications distributed non-randomly.

Structured ${B}_{ijk}$ arrays were generated by first identifying a set of trophic interaction modifications according to the ‘mutual interference’ model described above at a frequency of $0.1{\mathrm{N}}^{2}$, then inferring the generated higher-order effects. To maintain average interaction strengths with potentially varying connectance, elements of ${B}_{ijk}$ were initially assigned a value of +1 or −1, then all interaction arrays were first scaled to a variance of 1 before being rescaled by the strength terms ($\upbeta ,\upgamma$). Structured four-way ${C}_{ijkl}$ arrays were specified by first identifying interaction modifications in the same way to specify $i$, $j$ and $k$, then for the remaining vector ${C}_{[i,j,k, ]}$ defined by $l$, each possible four-way interaction occurred with a 10% probability.

## Results

### Impact on community structure

**Fig. 4:**
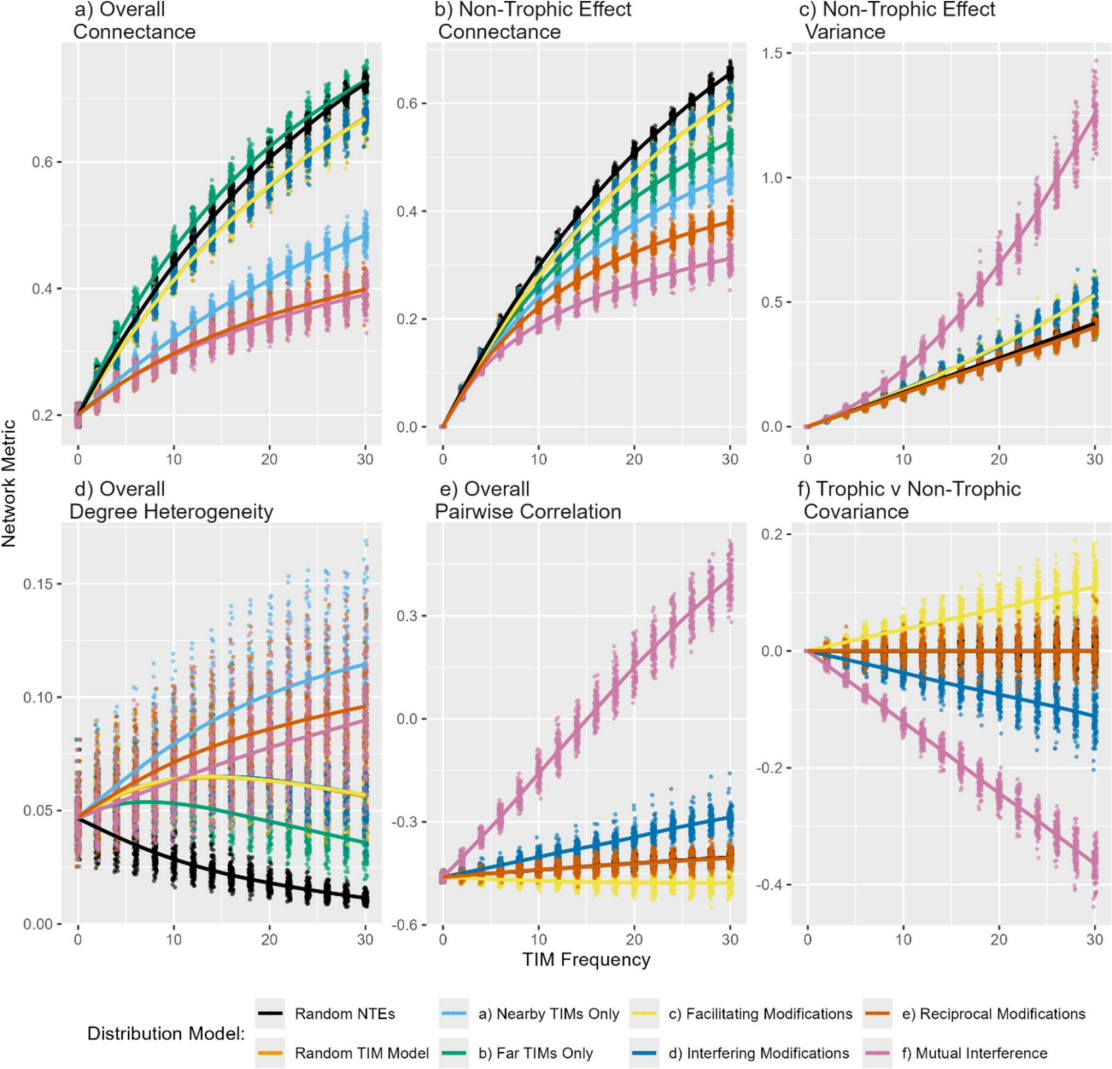
Impact of different distributions of non-trophic effects on network structures. **a** Connectance (fraction non-zero entries) of overall interaction matrix $\mathbf{A}$. **b** Connectance of non-trophic interaction matrix $\mathbf{C}$. **c** Variance of the off-diagonal non-trophic elements $\mathbf{C}$. **d** Degree heterogeneity of $\mathbf{A}$ (variance of the normalised in and out-degree distribution. **e** Correlation of the pairwise elements within $\mathbf{A}: {\left({\mathrm{A}}_{ij},{\mathrm{A}}_{ji}\right)}_{\mathrm{i}\ne \mathrm{j}}$. **f** Covariance between elements of trophic and non-trophic effects ($\mathrm{cov}(\mathbf{B},\mathbf{C})$). Colours denote different distributional models, lines are loess fits through 100 replicates at each TIM frequency

Different distributional models had highly distinct impacts on the structure of the network compared to the baseline, random, models (Fig. 4). Compared to random NTEs, the structured models could increase overall connectance faster (under the ‘Far’ TIM model) but for most the increase was considerably slower (Fig. 4a). The variance in NTE elements increased steadily with an increasing number of TIMs for most models but rose faster when the signs were fixed (facilitating/interfering-only models) and especially so under the mutual-interference model (Fig. 4c). High frequencies of mutual-interference TIMs had a dramatic effect on the pairwise correlation within the overall interaction matrix, pushing it positive at high frequencies of modifications (Fig. 4e). The most complex patterns were seen in degree-heterogeneity (Fig. 4d)—while random NTEs had a marked smoothing effect, the various structured models initially increased the variance in the number of interaction partners of each species. While most TIM distribution models did result in a covariance between trophic and non-trophic effects, those that introduced signed effects had marked effects (Fig. 4f) stemming from the distinct patterning at either the top or base of the trophic network (Figure S1).

### Impact on dynamics

**Fig. 5:**
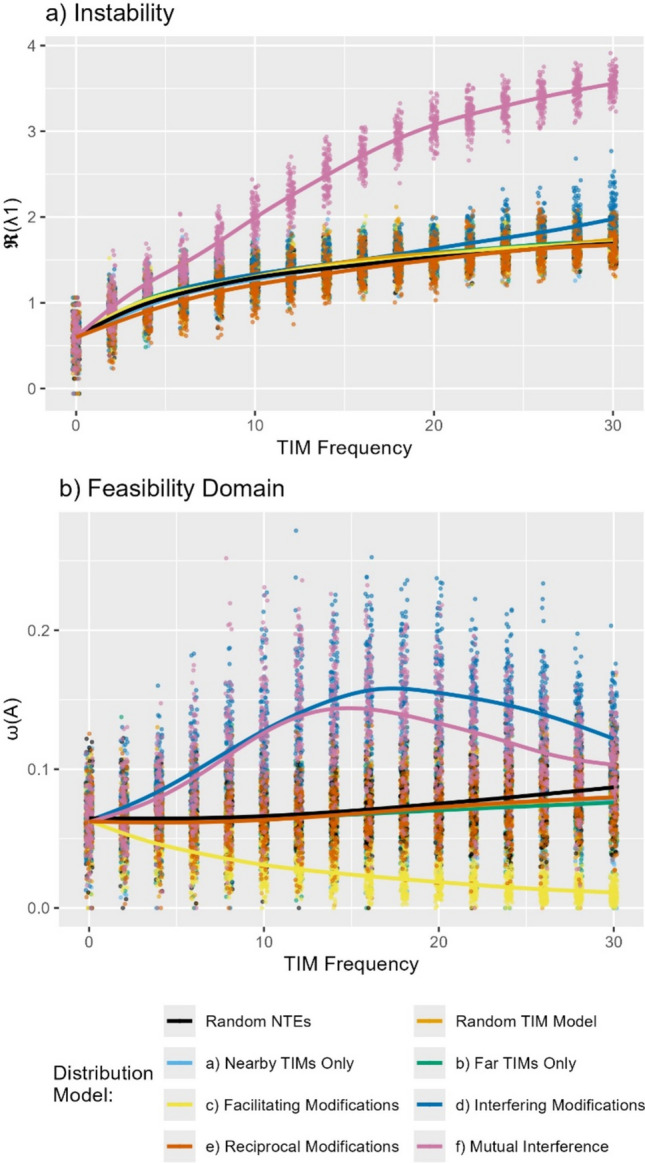
Impact of structured non-trophic effects on dynamic properties. **a** Effect of increasing frequency of TIMs on instability $\mathrm{log}\left(\mathfrak{R}\left({\uplambda }_{1}^{\mathbf{A}}\right)\right)$, the degree of self-regulation necessary for local asymptotic stability. **b** The size of the feasibility domain $\upomega \left(\mathbf{A}\right)$. Loess fitted lines have been added to highlight differences

Across our measurements of system dynamics, we found a distinct split between TIM distributions that had notably divergent effects and those where impacts were barely distinguishable from random non-trophic effects (Fig. 5). As the frequency of TIMs increased, local stability always decreased (Fig. 5a). Most distributional models were not distinct to the random baseline models, except for the mutual-interference model that has a strongly destabilising impact. As such, the topology of the TIMs interacted with sign-based effects—the mutual interference model effectively combines the ‘interfering’ and ‘reciprocal’ models, that did not have individual impacts on local stability.

By contrast, the response of the size of the feasibility domain as more TIMs were introduced was more divergent between models (Fig. 5b). Interfering TIMs increased the size of the feasibility domain, while facilitatory modifications had a negative effect. The TIM distribution models that solely change the topology of the TIM network relative to the food web, did not have a major difference from randomly distributed non-trophic effects. The results were qualitatively similar for alternative choices of the self-regulation term (SI, Figure S2).

### Contribution of higher-order interaction structure on diversity-stability relationship

**Fig. 6:**
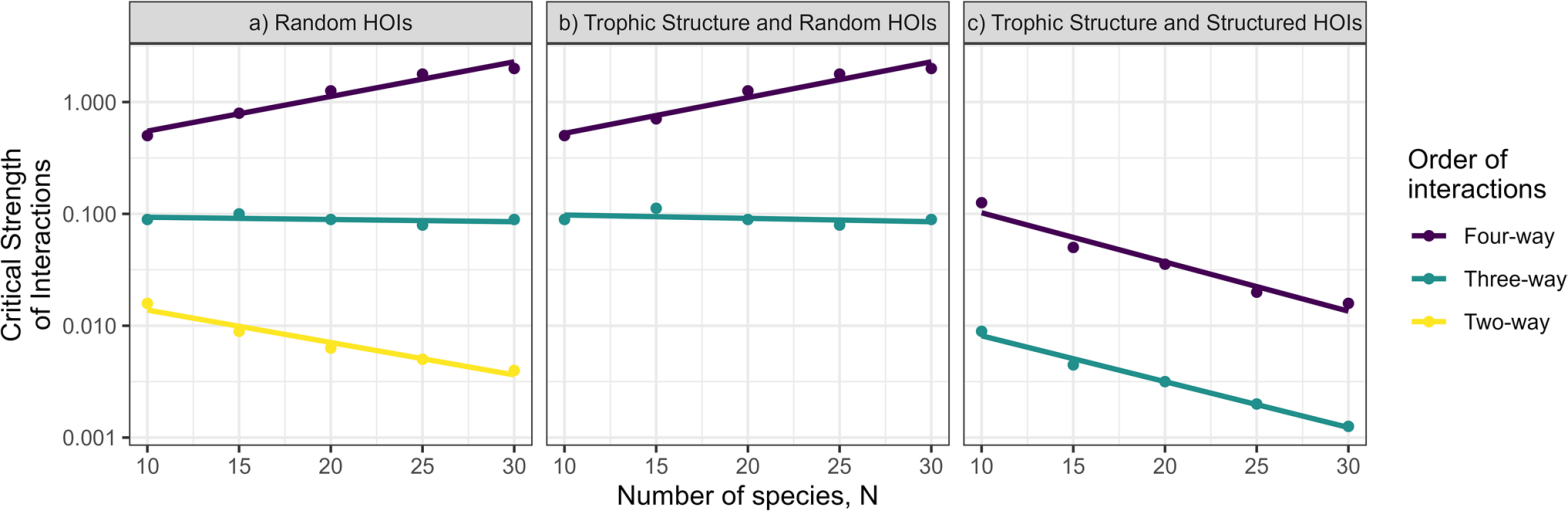
Dependence of critical strength of interactions on HOI order and distribution scenarios. Critical strength was the highest coefficient scaler ($\alpha$, $\upbeta$, $\upgamma$) where > 90% of 50 random communities showed no extinctions. a) Reproduction of results in Bairey et al. (2016), where interactions at each order (2, 3, 4) cause qualitatively different responses to increasing diversity. b) introducing an underlying trophic network structure with random HOIs maintains the original result c) Structured HOIs (3 or 4 way) reduce stability as diversity increases

Randomly distributed HOIs were found to nullify or reverse the pairwise diversity-stability relationship at 3rd and 4th order (Fig. 6a), reproducing earlier results (Bairey et al. 2016). The introduction of underlying trophic structure did not affect this pattern (Fig. 6b). However, when HOIs structured by the mutual interference distribution model are incorporated, the direction of the diversity-stability response was negative for both 3- and 4-way interactions (Fig. 6c).

## Discussion

Our results demonstrate that the structure of higher-order interactions can have strong impacts on the dynamics of food webs. Given that the study of the consequences of structured pairwise interaction networks has been a mainstay of ecological research for many decades (Dunne and Pascual 2006), this should not necessarily be surprising. However, consideration of structured higher-order interactions within larger communities has been notably slow to develop beyond the random case. These results identify how the combined effects of both topology, sign structure and relationship to pairwise interactions will be central to understanding their community-level consequences. Higher-order interactions have been previously identified as a generic potential source of stabilisation (Bairey et al. 2016; Grilli et al. 2017b). However, our observation that plausible structures can reverse a previously identified stabilising effect implies these results are a consequence of assuming unstructured HOIs distributions that tend to average out, rather than build on each other.

Trophic interaction modifications have the potential to cause significant disruptive effects upon the pattern of interactions within ecological communities that can influence system-level dynamics. The inter-relationship between classes of interaction is critical to understanding their consequences, reiterating the importance of the dependencies between superimposed interaction networks (Kéfi et al. 2016; Pilosof et al. 2017). Interaction modifications can influence the stability of systems beyond introducing additional connectance. They can shift the average interaction sign, change pairwise correlation coefficients, introduce additional row structure and change the interaction strength distribution.

Our results highlight the particular importance of HOI sign effects within trophic networks, in line with results from horizontal networks (Gibbs et al. 2024; Singh and Baruah 2021). Every interaction modification introduces two non-trophic effects, one positive, one negative. As such, they can be ‘signed’ in multiple ways. Firstly, each interaction modification is either interfering (weakening an interaction, resulting in a positive effect on the resource and a negative effect on the consumer) or facilitatory with the opposite effect. Furthermore, each pair of non-trophic effects can be unbalanced, with either greater positive or negative effects. Across the community, this could have a considerable impact on the overall sign distribution and dynamics. An empirical understanding of the distribution of these sign effects in real ecosystems is currently lacking, but our simulations suggest their balance could be central to understanding their impact at a system level.

Our results demonstrate how sign effects can be greatly magnified if they are also topologically structured. The most complex distribution model used here, reciprocal negative effects between resources that share a consumer, was designed to represent a structure likely to be particularly prevalent. It can be generated by a range of mechanisms, including predator satiation (Jeschke et al. 2004), adaptive foraging (Abrams 2010; Valdovinos et al. 2010) and associational defence (Barbosa et al. 2009). These effects are widespread (Bruno et al. 2003) and are regularly included in general models of population dynamics (Delmas et al. 2017) through multi-species functional responses (Koen-Alonso 2007), yet the resultant dynamic linkages are rarely considered in network-based analyses. It is therefore striking that the mutual interference model of TIM distributions, which represent these effects, has some of the most marked and complex impacts upon the dynamics of our systems. It causes strong local destabilisation, despite the lower variance in overall interaction strength (since negative non-trophic interactions overlap with positive trophic interaction terms), driven largely by emergent pairwise mutualism, long-recognized as destabilising for interaction matrices (May 1973). The NTEs induced by TIMs structured in this way are very efficient at breaking down the negative correlation between pairwise elements (Tang et al. 2014), inducing apparent mutualistic effects between resources that share a consumer. The mutual interference tends to focus negative and positive NTEs on to distinct groups of species (the high-level consumers and low-level resources). The importance of the sign structure of interference can also be seen in the comparative lack of distinction of the tightly reciprocal interaction modification distribution (Fig. 3d) or the interfering modification models—it is the specific sign patterning of the links that drives the change to the dynamics. In terms of the size of the feasibility domain, the close match with the randomly distributed interfering TIM model suggests that this effect is largely driven by sign, rather than interactions with topology.

The distribution of non-trophic effects in real communities, including those caused by interaction modifications, is at present essentially unknown beyond surveys from a limited number of inter-tidal communities (Kéfi et al. 2015; Sander et al. 2015) and model systems (Barbosa et al. 2023; Buche et al. 2024; Vandermeer and Perfecto 2024). The fraction of interspecific interactions driven by interaction modifications is unknown, but likely to be large (Abrams 1983; Werner and Peacor 2003). The set of models used here attempts to represent some of the properties real distributions of interaction modifications could have and identify features pertinent to dynamics. However, it can only be a stepping stone from fully random approaches towards real systems. It must be noted that the distribution of interaction strengths within real networks differs significantly from our randomly generated networks (Jacquet et al. 2016). They include both significant row-structuring and often show an approximately log-normal interaction trophic strength distribution.

Ideally, empirical data will need to include information about both the topology and strength of interaction modifications. Whilst it is unlikely that there will be strong mechanistic drivers of non-trophic network structure equivalent to the role of body size within trophic interaction networks (Brose 2010; Pawar et al. 2012; Petchey et al. 2008), there is nevertheless room for a great improvement in our understanding of the distribution of interaction modifications. There may be scope to develop broad categorisations of functional groups of species that cause, or respond to, changing biotic environments affecting consumption rates. A related area in need of progress is the development of consistent terminology and metrics to describe the structure of multiplex interaction networks that span several orders. This problem is not unique to ecology (Aleta and Moreno 2019; Battiston et al. 2021; Majhi et al. 2022), but ecologists are likely to be interested in distinct summaries of these complex structures and need a vocabulary that can bridge between theory and empiricists.

Analytically identifying the unique ‘dynamic’ contribution of higher-order effects (distinct to their effective pairwise effects) is not straightforward as differences will arise away from any equilibrium, over varying timescales. Collapsing higher-order effects into effective pairwise interactions can help understand the impact on near-equilibrium dynamics but does not necessarily capture responses to larger disruptions (e.g. Letten and Stouffer 2019; Terry et al. 2019). We are currently limited to either trialling the effect of potential HOI distributions (this study), identifying features of particularly influential interaction modifications (Terry et al. 2020) or exploring the properties of HOI structures that can contribute to stability (Gibbs et al. 2024). All will ultimately require a firmer foundation in empirical data, but this need not prevent the exploration of alternatives.

Given the potential for interaction modifications to short-circuit established trophic interaction motifs such as tri-trophic cascades, the distribution of interaction motifs in ecological communities (Milo et al. 2002; Stouffer et al. 2007) may need to be re-examined to incorporate non-trophic effects that emerge. As a first step, our results suggest that assessments of the sign-balance of trophic interaction modifications could be highly informative about their impact in natural systems. The potential impact of HOIs on community persistence may come down to a simpler question—will multiple effective impacts of higher-order interactions stack or cancel out?

## Conclusion

Interaction modifications within trophic networks offer one of the few scenarios where plausible guesses can be made about the possible distribution of higher-order interactions within ecological communities. We have shown that they can have a significant impact on the structure and dynamics of ecological communities and reverse diversity-stability relationships. While further data will be central, research should pay closer attention to the consequences of assuming unstructured higher-order interactions.

## Supplementary Information

Below is the link to the electronic supplementary material.

Supplementary file1 (DOCX 600 KB)

## Data Availability

R code and generated data are available at github.com/jcdterry/TIMStructure_Stability_Public and archived on Zenodo at 10.5281/zenodo.13842454.

## References

[CR1] Abrams PA (1983) Arguments in favor of higher order interactions. Am Nat 121:887–891

[CR2] Abrams PA (2001) Describing and quantifying interspecific interactions: a commentary on recent approaches. Oikos 94:209–218

[CR3] Abrams PA (2010) Implications of flexible foraging for interspecific interactions: lessons from simple models. Funct Ecol 24:7–17

[CR4] AlAdwani M, Saavedra S (2019) Is the addition of higher-order interactions in ecological models increasing the understanding of ecological dynamics? Math Biosci 315:108222 31260670 10.1016/j.mbs.2019.108222

[CR5] Aleta A, Moreno Y (2019) Multilayer networks in a nutshell. Ann Rev Condens Matter Phys 10:45–62

[CR6] Allesina S, Grilli J, Barabás G, Tang S, Aljadeff J, Maritan A et al (2015) Predicting the stability of large structured food webs. Nat Commun 6:7842 26198207 10.1038/ncomms8842 PMC4525179

[CR7] Bairey E, Kelsic ED, Kishony R (2016) High-order species interactions shape ecosystem diversity. Nat Commun 7:12285 27481625 10.1038/ncomms12285 PMC4974637

[CR8] Barabás G, Michalska-Smith MJ, Allesina S (2017) Self-regulation and the stability of large ecological networks. Nat Ecol Evol 1:1870–1875 29062124 10.1038/s41559-017-0357-6

[CR9] Barbosa P, Hines J, Kaplan I, Martinson H, Szczepaniec A, Szendrei Z (2009) Associational resistance and associational susceptibility: having right or wrong neighbors. Annu Rev Ecol Evol Syst 40:1–20

[CR10] Barbosa M, Fernandes GW, Morris RJ (2023) Experimental evidence for a hidden network of higher-order interactions in a diverse arthropod community. Curr Biol 33:381-388.e4 36563693 10.1016/j.cub.2022.11.057

[CR11] Battiston F, Amico E, Barrat A, Bianconi G, Ferraz de Arruda G, Franceschiello B et al (2021) The physics of higher-order interactions in complex systems. Nat Phys 17:1093–1098

[CR12] Billick I, Case TJ (1994) Higher order interactions in ecological communities: what are they and how can they be detected? Ecology 75:1530–1543

[CR13] Bolker B, Holyoak M, Křivan V, Rowe L, Schmitz O (2003) Connecting theoretical and empirical studies of trait-mediated interactions. Ecology 84:1101–1114

[CR14] Brose U (2010) Body-mass constraints on foraging behaviour determine population and food-web dynamics. Funct Ecol 24:28–34

[CR15] Bruno JF, Stachowicz JJ, Bertness MD (2003) Inclusion of facilitation into ecological theory. Trends Ecol Evol 18:119–125

[CR16] Buche L, Bartomeus I, Godoy O (2024) Multitrophic higher-order interactions modulate species persistence. Am Nat 203:458–472 38489780 10.1086/729222

[CR17] Cohen JE (1978) Food Webs and Niche Space. Princeton University Press, Princeton

[CR18] Coyte KZ, Schluter J, Foster KR (2015) The ecology of the microbiome: networks, competition, and stability. Science 350:663–666 26542567 10.1126/science.aad2602

[CR19] Delmas E, Brose U, Gravel D, Stouffer DB, Poisot TT (2017) Simulations of biomass dynamics in community food webs. Methods Ecol Evol 8:881–886

[CR20] Donohue I, Petchey OL, Kéfi S, Génin A, Jackson AL, Yang Q et al (2017) Loss of predator species, not intermediate consumers, triggers rapid and dramatic extinction cascades. Glob Change Biol 23:2962–2972 10.1111/gcb.13703 28346736

[CR21] Dunne JA, Pascual M (2006) Ecological networks: linking structure to dynamics in food webs. Oxford University Press, Oxford

[CR22] Fontaine C, Guimarães PR, Kéfi S, Loeuille N, Memmott J, van der Putten WH et al (2011) The ecological and evolutionary implications of merging different types of networks. Ecol Lett 14:1170–1181 21951949 10.1111/j.1461-0248.2011.01688.x

[CR23] García-Callejas D, Molowny-Horas R, Araújo MB (2018) The effect of multiple biotic interaction types on species persistence. Ecology 99:2327–2337 30030927 10.1002/ecy.2465

[CR24] Gibbs T, Levin SA, Levine JM (2022) Coexistence in diverse communities with higher-order interactions. Proc Natl Acad Sci 119:e2205063119 36252042 10.1073/pnas.2205063119 PMC9618036

[CR25] Gibbs TL, Gellner G, Levin SA, McCann KS, Hastings A, Levine JM (2024) When can higher-order interactions produce stable coexistence? Ecol Lett 27:e14458 38877741 10.1111/ele.14458

[CR26] Golubski AJ, Westlund EE, Vandermeer J, Pascual M (2016) Ecological networks over the edge: hypergraph trait-mediated indirect interaction (TMII) structure. Trends Ecol Evol 31:344–354 26924738 10.1016/j.tree.2016.02.006

[CR27] Grilli J, Rogers T, Allesina S (2016) Modularity and stability in ecological communities. Nat Commun 7:12031 27337386 10.1038/ncomms12031 PMC4931019

[CR28] Grilli J, Adorisio M, Suweis S, Barabás G, Banavar JR, Allesina S et al (2017) Feasibility and coexistence of large ecological communities. Nat Commun 8:14389 28233768 10.1038/ncomms14389 PMC5333123

[CR29] Grilli J, Barabás G, Michalska-Smith MJ, Allesina S (2017) Higher-order interactions stabilize dynamics in competitive network models. Nature 548:210–213 28746307 10.1038/nature23273

[CR30] Ings TC, Montoya JM, Bascompte J, Blüthgen N, Brown L, Dormann CF et al (2009) Ecological networks-beyond food webs. J Anim Ecol 78:253–269 19120606 10.1111/j.1365-2656.2008.01460.x

[CR31] Jacquet C, Moritz C, Morissette L, Legagneux P, Massol F, Archambault P et al (2016) No complexity–stability relationship in empirical ecosystems. Nat Commun 7:12573 27553393 10.1038/ncomms12573 PMC4999500

[CR32] Jeschke JM, Kopp M, Tollrian R (2004) Consumer-food systems: why type I functional responses are exclusive to filter feeders. Biol Rev 79:337–349 15191227 10.1017/s1464793103006286

[CR33] Johnson S, Jones NS (2017) Looplessness in networks is linked to trophic coherence. Proc Natl Acad Sci 114:5618–5623 28512222 10.1073/pnas.1613786114 PMC5465891

[CR34] Kéfi S, Berlow EL, Wieters EA, Navarrete SA, Petchey OL, Wood SA et al (2012) More than a meal… integrating non-feeding interactions into food webs. Ecol Lett 15:291–300 22313549 10.1111/j.1461-0248.2011.01732.x

[CR35] Kéfi S, Berlow EL, Wieters EA, Joppa LN, Wood SA, Brose U et al (2015) Network structure beyond food webs : mapping non-trophic and trophic interactions on Chilean rocky shores. Ecology 96:291–303 26236914 10.1890/13-1424.1

[CR36] Kéfi S, Miele V, Wieters EA, Navarrete SA, Berlow EL (2016) How structured is the entangled bank? The surprisingly simple organization of multiplex ecological networks leads to increased persistence and resilience. PLoS Biol 14:e1002527 27487303 10.1371/journal.pbio.1002527 PMC4972357

[CR37] Kelsic ED, Zhao J, Vetsigian K, Kishony R (2015) Counteraction of antibiotic production and degradation stabilizes microbial communities. Nature 521:516–519 25992546 10.1038/nature14485 PMC4551410

[CR38] Kleinhesselink AR, Kraft NJB, Pacala SW, Levine JM (2022) Detecting and interpreting higher-order interactions in ecological communities. Ecol Lett 25:1604–1617 35651315 10.1111/ele.14022

[CR39] Koen-Alonso M (2007) A process-oriented approach to the multispecies functional response. In: Rooney N, McCann KS, Noakes DLG (eds) From energetics to ecosystems: the dynamics and structure of ecological systems. Springer, Netherlands, pp 1–36

[CR40] Křivan V (2014) Competition in di- and tri-trophic food web modules. J Theor Biol 343:127–137 24316384 10.1016/j.jtbi.2013.11.020

[CR41] Letten AD, Stouffer DB (2019) The mechanistic basis for higher-order interactions and non-additivity in competitive communities. Ecol Lett 22:423–436 30675983 10.1111/ele.13211

[CR42] Levine JM, Bascompte J, Adler PB, Allesina S (2017) Beyond pairwise mechanisms of species coexistence in complex communities. Nature 546:56–64 28569813 10.1038/nature22898

[CR43] Majhi S, Perc M, Ghosh D (2022) Dynamics on higher-order networks: a review. J R Soc Interface 19:20220043 35317647 10.1098/rsif.2022.0043 PMC8941407

[CR44] May RM (1972) Will a large complex system be stable? Nature 238:413–414 4559589 10.1038/238413a0

[CR45] May RM (1973) Stability and complexity in model ecosystems. Princeton University Press, Princeton

[CR46] Mayfield MM, Stouffer DB (2017) Higher-order interactions capture unexplained complexity in diverse communities. Nat Ecol Evol 1:0062 10.1038/s41559-016-0062 28812740

[CR47] McCann K, Hastings A, Huxel GR (1998) Weak trophic interactions and the balance of nature. Nature 395:794–798

[CR48] McCann KS (2011) Food Webs. Monographs in population biology. Princeton University Press.

[CR49] Miele V, Guill C, Ramos-Jiliberto R, Kéfi S (2019) Non-trophic interactions strengthen the diversity—functioning relationship in an ecological bioenergetic network model. PLoS Comput Biol 15:e1007269 31465440 10.1371/journal.pcbi.1007269 PMC6715155

[CR50] Milo R, Shen-Orr S, Itzkovitz S, Kashtan N, Chklovskii D, Alon U (2002) Network motifs: simple building blocks of complex networks. Science 298:824–827 12399590 10.1126/science.298.5594.824

[CR51] Montoya JM, Pimm SL, Solé RV (2006) Ecological networks and their fragility. Nature 442:259–264 16855581 10.1038/nature04927

[CR52] Moore JC, de Ruiter PC (2012) Energetic food webs: an analysis of real and model ecosystems. Oxford University Press

[CR53] Moore JC, de Ruiter PC, McCann KS, Wolters V (eds) (2017) Adaptive food webs: stability and transitions of real and model ecosystems. Cambridge University Press, Cambridge

[CR54] Neutel A-M, Heesterbeek JAP, de Ruiter PC (2002) Stability in real food webs: weak links in long loops. Science 296:1120–1123 12004131 10.1126/science.1068326

[CR55] Novak M, Yeakel JD, Noble AE, Doak DF, Emmerson M, Estes JA et al (2016) Characterizing species interactions to understand press perturbations: what is the community matrix? Annu Rev Ecol Evol Syst 47:409–432

[CR56] Ohgushi T, Schmitz O, Holt RD (2012) Trait-mediated indirect interactions: ecological and evolutionary perspectives. Cambridge University Press, Cambridge

[CR57] Olff H, Alonso D, Berg MP, Eriksson BK, Loreau M, Piersma T et al (2009) Parallel ecological networks in ecosystems. Philos Trans R Soc Lond B Biol Sci 364:1755–1779 19451126 10.1098/rstb.2008.0222 PMC2685422

[CR58] Pawar S, Dell AI, Savage VM (2012) Dimensionality of consumer search space drives trophic interaction strengths. Nature 486:485–489 22722834 10.1038/nature11131

[CR59] Petchey OL, Beckerman AP, Riede JO, Warren PH (2008) Size, foraging, and food web structure. Proc Natl Acad Sci 105:4191–4196 18337512 10.1073/pnas.0710672105 PMC2393804

[CR60] Pilosof S, Porter MA, Pascual M, Kéfi S (2017) The multilayer nature of ecological networks. Nat Ecol Evol 1:0101 10.1038/s41559-017-0101 28812678

[CR61] Poisot T, Baiser B, Dunne JA, Kéfi S, Massol F, Mouquet N et al (2016) Mangal–making ecological network analysis simple. Ecography 39:384–390

[CR62] Preisser EL, Bolnick DI, Benard MF (2005) Scared to death? The effects of intimidation and consumption in predator-prey interactions. Ecology 86:501–509

[CR63] Pringle RM, Kartzinel TR, Palmer TM, Thurman TJ, Fox-Dobbs K, Xu CCY et al (2019) Predator-induced collapse of niche structure and species coexistence. Nature 570:58–64 31168105 10.1038/s41586-019-1264-6

[CR64] Rohr RP, Saavedra S, Bascompte J (2014) On the structural stability of mutualistic systems. Science 345:1253497–1253497 25061214 10.1126/science.1253497

[CR65] Saavedra S, Rohr RP, Bascompte J, Godoy O, Kraft NJB, Levine JM (2017) A structural approach for understanding multispecies coexistence. Ecol Monogr 87:470–486

[CR66] Sander EL, Wootton JT, Allesina S (2015) What can interaction webs tell us about species roles? PLoS Comput Biol 11:e1004330 26197151 10.1371/journal.pcbi.1004330 PMC4511233

[CR67] Sanders D, Jones CG, Thébault E, Bouma TJ, Heide TVD, Belzen JV et al (2014) Integrating ecosystem engineering and food webs. Oikos 123:513–524

[CR68] Singh P, Baruah G (2021) Higher order interactions and species coexistence. Thyroid Res 14:71–83

[CR69] Song C, Rohr RP, Saavedra S (2018) A guideline to study the feasibility domain of multi-trophic and changing ecological communities. J Theor Biol 450:30–36 29702110 10.1016/j.jtbi.2018.04.030

[CR70] Stouffer DB, Camacho J, Jiang W, Amaral LAN, Nunes Amaral LA (2007) Evidence for the existence of a robust pattern of prey selection in food webs. Proc R Soc B Biol Sci 274:1931–1940 10.1098/rspb.2007.0571 PMC2275185 17567558

[CR71] Suraci JP, Clinchy M, Dill LM, Roberts D, Zanette LY (2016) Fear of large carnivores causes a trophic cascade. Nat Commun 7:10698 26906881 10.1038/ncomms10698 PMC4766389

[CR72] Tang S, Pawar S, Allesina S (2014) Correlations between interation strengths drives stability in large ecological networks - Supp Info. Ecol Lett 17:1094–1100 24946877 10.1111/ele.12312

[CR73] Terry JCD, Morris RJ, Bonsall MB (2017) Trophic interaction modifications: an empirical and theoretical framework. Ecol Lett 20:1219–1230 28921859 10.1111/ele.12824 PMC6849598

[CR74] Terry JCD, Morris RJ, Bonsall MB (2019) Interaction modifications lead to greater robustness than pairwise non-trophic effects in food webs. J Anim Ecol 1365–2656:13057 10.1111/1365-2656.13057 PMC6900167 31287921

[CR75] Terry JCD, Bonsall MB, Morris RJ (2020) Identifying important interaction modifications in ecological systems. Oikos 129:147–157

[CR76] Thébault E, Fontaine C (2010) Stability of ecological communities and the architecture of mutualistic and trophic networks. Science 329:853–856 20705861 10.1126/science.1188321

[CR77] Valdovinos FS, Ramos-Jiliberto R, Garay-Narváez L, Urbani P, Dunne JA (2010) Consequences of adaptive behaviour for the structure and dynamics of food webs. Ecol Lett 13:1546–1559 20937057 10.1111/j.1461-0248.2010.01535.x

[CR78] van Veen FJF, van Holland PD, Godfray HCJ (2005) Stable coexistence in insect communities due to density- and trait-mediated indirect effects. Ecology 86:3182–3189

[CR79] Vandermeer J, Perfecto I (2024) Combining intransitive and higher-order effects in a coupled oscillator framework: a case study of an ant community. Ecology 105:e4218 38032663 10.1002/ecy.4218

[CR80] Werner EE, Peacor SD (2003) A review of trait-mediated indirect interactions in ecological communities. Ecology 84:1083–1100

[CR81] Williams RJ, Martinez ND (2000) Simple rules yield complex food webs. Nature 404:180–183 10724169 10.1038/35004572

[CR82] Williams RJ, Martinez ND (2008) Success and its limits among structural models of complex food webs. J Anim Ecol 77:512–519 18284474 10.1111/j.1365-2656.2008.01362.x

[CR83] Wilson DS (1992) Complex interactions in metacommunities, with implications for biodiversity and higher levels of selection. Ecology 73:1984–2000

[CR84] Wootton JT (1994) Putting the pieces together: testing the independence of interactions among organisms. Ecology 75:1544–1551

[CR85] Wootton JT (1994) The nature and consequences of indirect effects in ecological communities. Annu Rev Ecol Syst 25:443–466

[CR86] Yodzis P (2000) Diffuse effects in food webs. Ecology 81:261–266

