# Supplementary material for: The impact of structured higher-order interactions on ecological network stability
Source: Theor Ecol. 2025 Jan 29;18(1):9. doi: 10.1007/s12080-025-00603-0 (PMC12799688; doi:10.1007/s12080-025-00603-0)
Supplement: Supplementary file 1 — Supplementary file1 (DOCX 600 KB) [file 12080_2025_603_MOESM1_ESM.docx]

**SI Figures
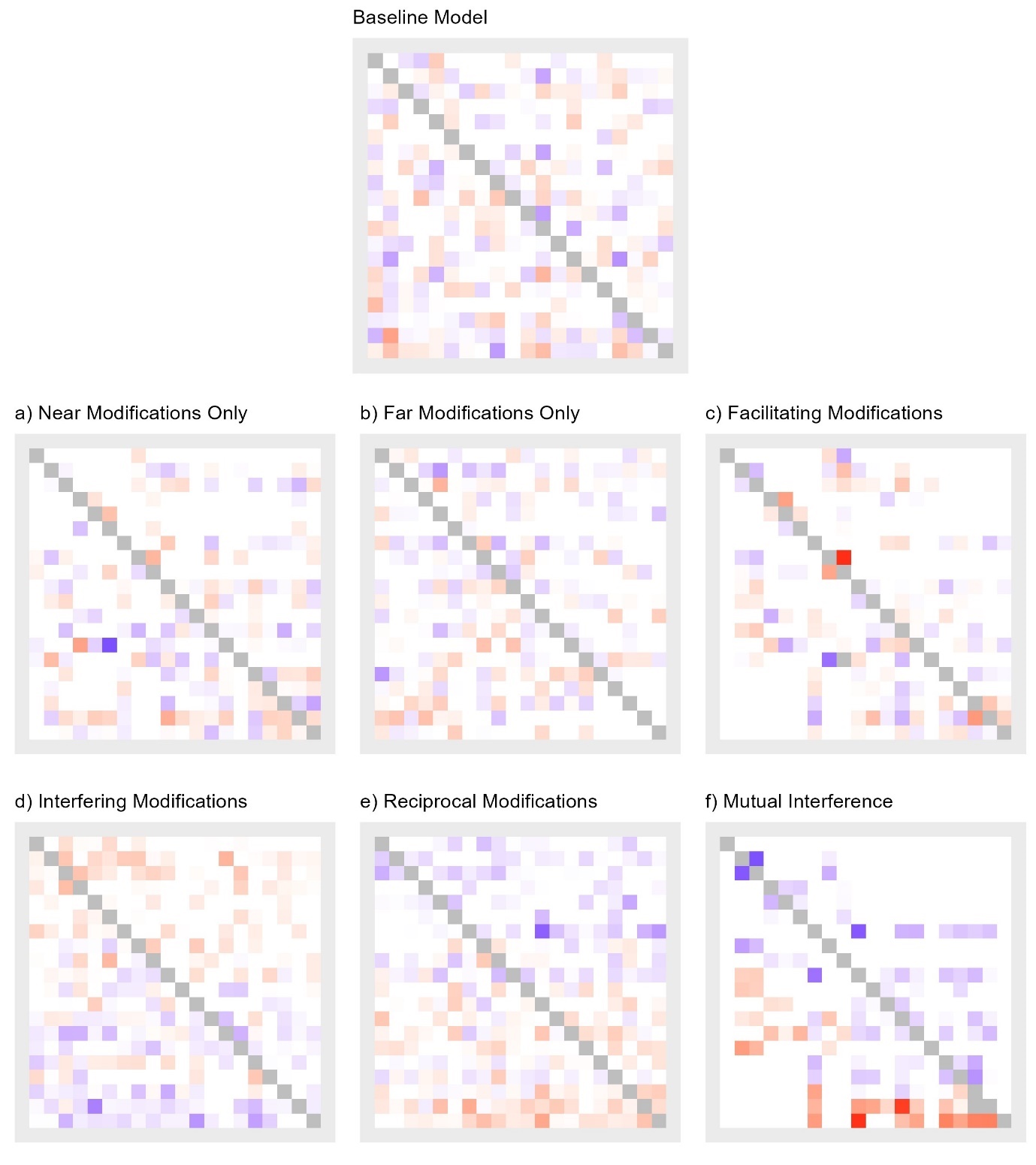
**

**Figure S1** Example NTE matrices generated from the different interaction modification distribution models, on top of the same underlying food web. Each entry represents the total interaction between two species - red colours indicate negative, the blue positive, with the intensity of the total indicating the magnitude.

**
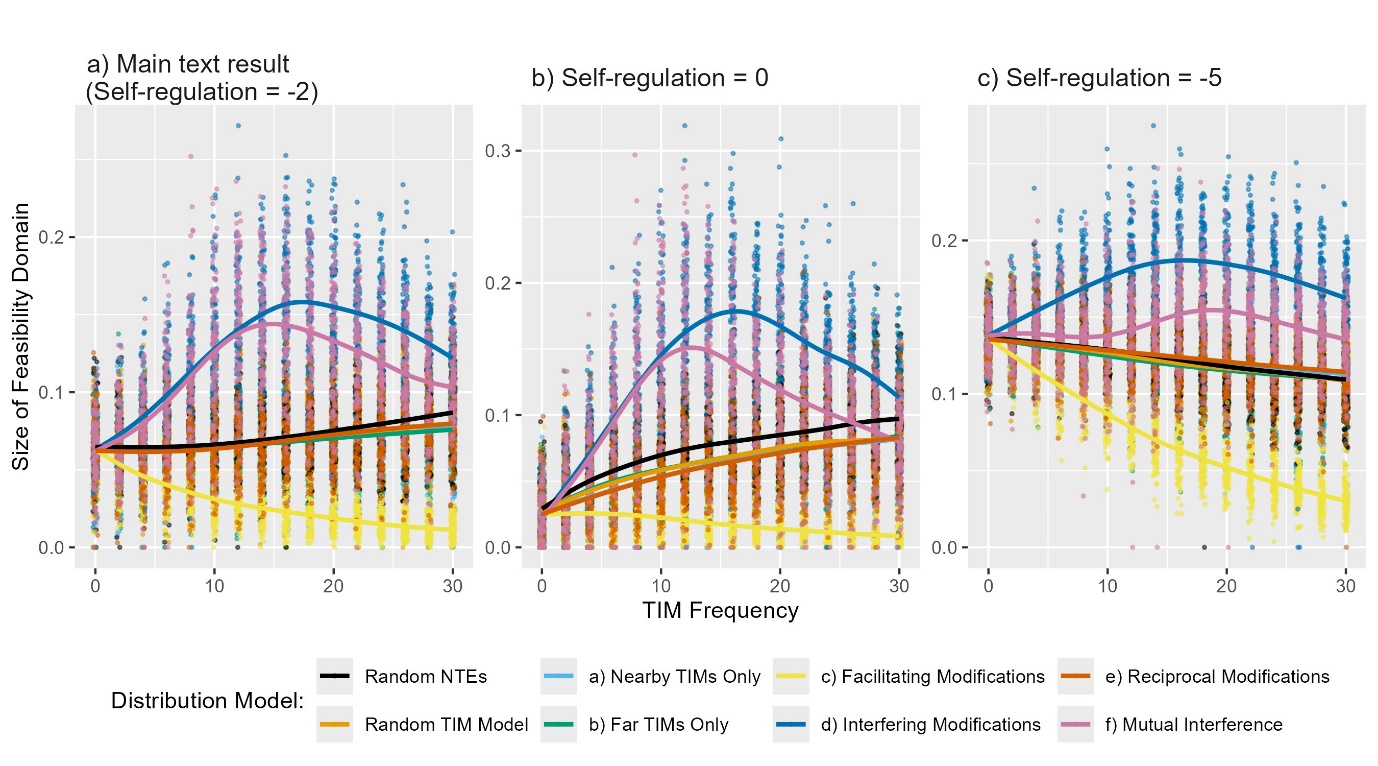
**

**Figure S2** Comparison of response of the size of the feasibility domain to increasing TIM frequency under different assumptions of the self-regulation (the diagonal terms of the equilibrium community matrix). Increasing self-regulation can alter the response, but the overall pattern of different distributions remains similar.
